# Current Updates on Involvement of Artificial Intelligence and Machine Learning in Semen Analysis

**DOI:** 10.3390/medicina60020279

**Published:** 2024-02-06

**Authors:** Manesh Kumar Panner Selvam, Ajaya Kumar Moharana, Saradha Baskaran, Renata Finelli, Matthew C. Hudnall, Suresh C. Sikka

**Affiliations:** 1 Department of Urology, Tulane University School of Medicine, New Orleans, LA 70112, USA; amoharana@tulane.edu (A.K.M.); saradhabaskaran@gmail.com (S.B.); ssikka@tulane.edu (S.C.S.); 2 Redox Biology & Proteomics Laboratory, Department of Zoology, School of Life Sciences, Ravenshaw University, Cuttack 753003, Odisha, India; 3 CREATE Fertility, 150 Cheapside, London EC2V 6ET, UK; finellirenata@gmail.com; 4 Cryobio and Reproductive Diagnostics, Inc., Columbus, OH 43214, USA; matthew.hudnall@cryobio.com

**Keywords:** artificial intelligence, machine learning, deep learning, semen, sperm

## Abstract

*Background and Objectives*: Infertility rates and the number of couples undergoing reproductive care have both increased substantially during the last few decades. Semen analysis is a crucial step in both the diagnosis and the treatment of male infertility. The accuracy of semen analysis results remains quite poor despite years of practice and advancements. Artificial intelligence (AI) algorithms, which can analyze and synthesize large amounts of data, can address the unique challenges involved in semen analysis due to the high objectivity of current methodologies. This review addresses recent AI advancements in semen analysis. *Materials and Methods*: A systematic literature search was performed in the PubMed database. Non-English articles and studies not related to humans were excluded. We extracted data related to AI algorithms or models used to evaluate semen parameters from the original studies, excluding abstracts, case reports, and meeting reports. *Results*: Of the 306 articles identified, 225 articles were rejected in the preliminary screening. The evaluation of the full texts of the remaining 81 publications resulted in the exclusion of another 48 articles, with a final inclusion of 33 original articles in this review. *Conclusions*: AI and machine learning are becoming increasingly popular in biomedical applications. The examination and selection of sperm by andrologists and embryologists may benefit greatly from using these algorithms. Furthermore, when bigger and more reliable datasets become accessible for training, these algorithms may improve over time.

## 1. Introduction

Infertility is defined as the failure to achieve a pregnancy despite engaging in regular, unprotected intercourse for at least a year [1,2]. While this condition affects more than 80 million couples in the reproductive age group worldwide, the male factor is reportedly responsible for approximately 50% of all infertility cases [3,4]. Laboratory evaluation of male infertility is currently performed by analyzing fresh semen specimens as per the WHO guidelines [2]. Such evaluations include examining both macroscopic (semen volume, viscosity, consistency, pH) and microscopic (sperm concentration, motility, morphology, and other cellular components) elements. Despite years of practice and advances, standard semen analysis still has high variability with relatively low accuracy and specificity [5]. Furthermore, inter- and intra-observer variations also affect the semen analysis results, mainly as a result of not fully adhering to the WHO guidelines [3,6]. There are significant challenges in performing standardized semen analysis, conducting vigorous training, and comparing results across various laboratories [4]. In an attempt to overcome such limitations and to better standardize the analysis, numerous semi- and completely automated computer-assisted semen analysis (CASA) systems have been developed. However, while automation of the semen analysis process is desirable, CASA systems are still far from being perfect, particularly due to the inaccurate identification of spermatozoa from other seminal components of comparable size, such as spherical cells, cytoplasmic droplets, or other debris, which represent the main challenges [4,7].

The ability to simulate human cognitive processes using a machine is known as artificial intelligence (AI). AI can perform jobs more effectively than humans with the clever integration of computer science, algorithms, machine learning (ML), and data sciences [8]. In neural-network-based ML, algorithms are created where machines learn and solve problems like the human mind [9]. AI-based approaches are currently used to generate real-time estimations of health risks for the diagnosis of various diseases, including skin, liver, and heart diseases, as well as Alzheimer’s [10], in an effort to minimize errors in medical practice involving diagnosis and treatment [11,12]. Similarly, the use of ML in laboratory practices is gaining popularity. A simple example of ML utilized in the laboratory is linear regression analysis, which forecasts standard instrument calibration [13]. These developments hold promise for laboratory testing, serving as a major foundation for clinical decision making [14].

Although AI technology has been evolving since the 1950s [15], it was first applied to male reproductive health in the early 2010s [16]. As AI becomes increasingly popular, algorithms are being trained and developed using data on age, abstinence period, semen parameters, cigarette smoking, and hematological status to predict sperm DNA damage and infertility conditions such as azoospermia [17,18,19]. Similarly, ML algorithms combined with digital holographic microscopy were used to assess the impact of oxidative damage on sperm motility and morphology [20]. In recent years, there has been a significant increase in the usage of AI, particularly in the analysis of sperm morphology and motility, as well as in improving the selection of the most appropriate sperm cells for use in assisted reproductive technology (ART) procedures [12]. Our current systematic review aims to discuss such involvement of AI and its potential applications in advancing our knowledge in andrology laboratory procedures such as semen analysis, detecting sperm DNA integrity, and predicting the success of surgical testicular sperm extraction (TESE).

## 2. Materials and Methods

**Figure 1:**
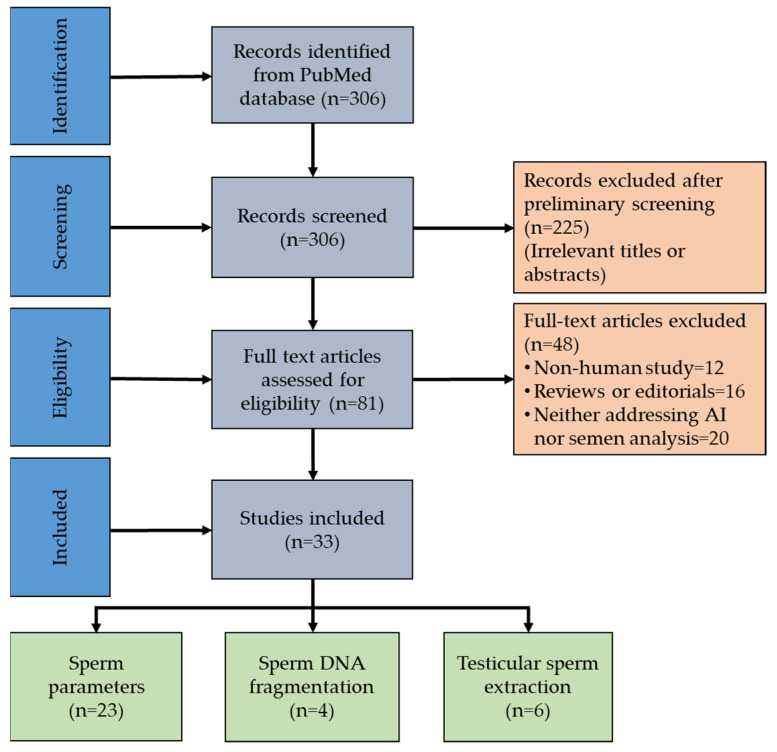
Preferred Reporting Items for Systematic Reviews and Meta-Analysis flow diagram of study identification and selection.

We conducted a systematic literature search in PubMed, following the PRISMA (Preferred Reporting Items for Systematic Reviews and Meta-Analysis) guidelines [21]. The search was limited to scientific articles published until July 2023. The following keywords were used to retrieve the articles: (semen OR semen quality OR semen parameters OR semen analysis OR sperm morphology OR sperm motility OR sperm viability OR sperm selection) AND (artificial intelligence OR machine learning OR deep learning). Figure 1 explains the steps involved in the identification of relevant articles. We extracted these data from the original studies while excluding abstracts, case reports, and meeting reports. Non-English articles and studies not related to humans were also excluded. The retrieved articles were screened for title and abstract independently by two authors. Furthermore, the full-text articles were evaluated for eligibility based on predefined inclusion criteria. To be considered for inclusion, studies had to discuss any type of AI method (such as machine learning, neural networks, deep learning, etc.) or perform the full or semi-autonomous analysis of any semen parameter or sperm DNA integrity or predict success of TESE. Studies involving only AI or ML and not focusing on semen parameters were excluded. Similarly, studies on semen parameters not highlighting the involvement of either form of AI or ML were also excluded.

## 3. Results

A total of 306 papers were identified after a thorough literature evaluation using PubMed database search. Eighty-one publications were selected after initial screening; these included various AI applications used to evaluate semen parameters. The evaluation of the full texts resulted in the exclusion of another 48 publications, with a final inclusion of 33 original articles in this systematic review (Figure 1, Supplementary Table S1).

## 4. Discussion

### 4.1. AI in Evaluation of Sperm Concentration or Total Count

Poor semen quality with reduced sperm concentration or total count is linked with a majority of male infertility cases. Hemocytometry, microfluidic techniques, spectrophotometry, and CASA systems are common methods for measuring sperm concentration or count [22]. CASA is the most effective approach for analyzing semen in clinics due to its quick turnaround time in reporting the results [7,23]. On the other hand, CASA systems are expensive, with system-to-system variation in sperm image processing that adversely affects the results of estimated sperm concentration or count [24]. AI has become widely accepted in medical applications that can deal with big data and heterogeneous information [25]. Semen samples contain heterogeneous populations of sperm and other non-sperm cells. Hence, applying ML and AI tools in semen analysis may be useful in improving the accuracy of the results. Studies that used ML and AI tools to assess sperm concentration or count are listed in Table 1.

AI tools can predict sperm concentration or count with the highest accuracy of 90%, among other seminal parameters [25]. Advanced ML algorithms also demonstrated a good correlation among semen parameters (total sperm count and total motile sperm) reported automatically using AI compared to manual evaluation [27]. Recently, an artificial neural network (ANN) algorithm was used to forecast the outcomes of a semen analysis. An FSNN (full-spectrum neural network) model, an ANN-based spectrophotometry methodology which is a quick, inexpensive, and effective, could predict sperm concentration with an accuracy of 93%, with significant positive correlation (R^2^ = 0.98, *p* ≤ 0.05) with clinical data [23]. AI-based models were also developed to estimate the possibility of an increase in sperm concentration following varicocele repair [26].

### 4.2. AI in Evaluation of Sperm Motility

The sperm motility and kinematics results reported by CASA systems are very reliable, as they show a high correlation with manual semen analysis. However, CASA systems fail to describe the motility or kinematics at the level of a single sperm [28,29]. Despite the automation of sperm motility analysis by CASA, their operational difficulties limit their widespread acceptance [30]. To overcome these problems, several researchers have used AI to categorize the motility of the whole sample as well as individual spermatozoa [17,29,31]. Table 2 summarizes the AI algorithms used to evaluate and predict total sperm motility.

Convolutional neural networks (CNNs) are notable AI algorithms developed by the integration of videotape recordings of sperm movement to categorize sperm motility into progressive, non-progressive, and immotile [31]. These CNN algorithms are also trained using sperm videos from the multinational video dataset of human spermatozoa (VISEM) [32,34]. Sperm head movement predicted by the R-CNN (region-based CNN) tracking algorithm had a very good positive correlation (r = 0.969) with laboratory analysis methods [34].

Besides CNNs, other ML models, such as support vector machine learning (SVM) and ANNs, are also well suited to tracking sperm motility. The SVM model classifies sperm motility as weak, non-vigorous, hyperactivated, intermediate, and progressive, with a predictive accuracy of 89% [29]. In contrast, the ANN model’s accuracy for evaluating total sperm motility is slightly lower than concentration [25]. Furthermore, AI-based image recognition of sperm motility coupled with a smartphone interface has been shown to accurately evaluate total motile sperm concentration (r = 0.84, *p* ≤ 0.05) and motility percentage (r = 0.90, *p* ≤ 0.05) [27].

In addition, AI plays an important role in accurately monitoring the characteristics of individual sperm. When coupled with additional ML models that examine sperm motility, there is a real possibility of automating the selection of a single excellent sperm for successful intra-cytoplasmic sperm injection (ICSI). Neural networks can improve the accuracy, speed, and dependability of automated single sperm identification procedures for ICSI, where intra- and inter-operator variation are unavoidable [28]. A faster region CNN (FR-CNN) was used to train a novel tail-to-head movement (THMA) algorithm to measure sperm motility, and its tracking capacity had a 97% success rate of accurately classifying individual sperm into low, moderate, and high motility [33].

### 4.3. AI in Evaluation of Sperm Morphology

Examining sperm morphology is one of the microscopic evaluations in a semen analysis that examines the form, shape, and size of spermatozoa [35]. Male fertility is significantly impacted by the morphological abnormalities of sperm [36]. It has proven difficult to standardize sperm morphology evaluation compared to other parameters such as concentration and motility. Several AI and ML-based models are being continuously trained using morphology characteristics as per WHO guidelines to classify spermatozoa as normal or abnormal [28]. AI-based morphology is evaluated based on the head dimensions alone or combined with other parts (mid-piece and tail) of spermatozoa [28]. Table 3 summarizes the AI algorithms used to analyze human sperm morphology. Two of the most commonly utilized AI algorithms for morphology analysis are deep neural networks (DNNs) and SVM. These AI algorithms and ML models were mainly trained using sperm images from different datasets such as Human Sperm Head Morphology (HuSHeM) [37], the gold-standard dataset for the morphological classification of human sperm heads (SCIAN) [38], and the Modified Human Sperm Head Morphology Analysis (MHSMA) [39].

The MHSMA database contains 1540 sperm images of the acrosome, head, neck, tail, and vacuole labeled as normal or abnormal. Deep learning algorithms have been trained using the MHSMA database to evaluate low-resolution sperm images. Furthermore, these algorithms allow the selection of the most appropriate fresh sperm cell (non-stained) for ICSI procedures with high accuracy [39]. Abbasi et al., 2021 proposed deep transfer learning (DTL) and deep multi-task transfer learning (DMTL) algorithms to automate the sperm morphology evaluation process with high accuracy using images (of the head, acrosome, neck, tail, and vacuole) from the MHSMA dataset [41].

The SCIAN-Morpho dataset contains a total of 1132 bright field images of normal as well as abnormal sperm. Furthermore, abnormal sperm head forms were divided into four sub-classes: small, tapering, amorphous, and pyriform [38]. CNN models, a K-means clustering approach, an SVM classifier, and a sequential forward feature selection (SFFS) algorithm were used to develop an automatic segmentation framework technique that validated sperm images from the SCIAN-SpermSegGS dataset with segmentation accuracies of 90%, 77%, and 78% for the head, acrosome, and nucleus, respectively [43]. The performances of deep learning architectures such as U-Net and Mask-RCNN were also assessed using images (210 sperm cells) from the SCIAN-SpermSegGS public dataset [35]. Riordon et al., 2019 reported that Visual Geometry Group 16 (VGG16), a deep CNN algorithm retrained using the HuSHeM and SCIAN datasets, provides better results (i.e., a true positive rate of 94%) compared to the CE-SVM approach [44]. In addition to VGG16, a special CNN architecture, Morphological Classification of Human Sperm Heads (MC-HSH), was proposed to accurately categorize sperm heads using the SCIAN-Morpho and HuSHeM datasets with an accuracy of 63% and 95%, respectively [47]. Shaker et al., 2017 applied an adaptive patch-based dictionary learning (APDL) approach to the SCIANMorphoSpermGS and HuSHeM datasets. The APDL algorithm classified human sperm head images as normal, tapered, pyriform, and amorphous with an accuracy of 92.2% and a precision of 93.5% [46].

Besides these, AI is also used in the stain-free morphological evaluation of both normal and abnormal sperm [20,42]. Nygate et al., 2020 generated holographic virtual stained sperm images and processed them using deep convolutional generative adversarial networks (DCGANs). Such virtual staining methods and DCGAN algorithms are valuable tools for researchers and physicians to accurately analyze morphology when chemical staining is not recommended for spermatozoa to be used for IVF or ICSI procedures [42]. Furthermore, Mirsky et al., 2017 used an SVM classifier to automate the sperm morphology (normal and abnormal) evaluation based on stain-free optical images acquired through interferometric phase microscopy (IPM) [45]. The YOLO v3 deep learning-based ML model detects abnormal sperm with a high sensitivity (0.881) and positive predictive value (0.853). In addition to morphological evaluation, this algorithm tracks the movement of sperm in a short time under an inverted microscope to select better-quality spermatozoa for ART procedures [40].

### 4.4. AI in Evaluation of Sperm DNA Integrity or Damage

A variety of laboratory tests such as single-cell gel electrophoresis or Comet assay, TUNEL (terminal deoxynucleotidyl transferase dUTP nick end labeling) assay, SCD (sperm chromatin dispersion) assay, SCSA (sperm chromatin structure assay), and acridine orange (AO) assay are available to measure the DNA integrity of spermatozoa [48]. The results obtained from these techniques display a high degree of inter- and intra-observer variation. AI algorithms might offer a new solution due to their effectiveness and dependability. DNA fragmentation index (DFI) values were predicted by employing deep learning methods using corresponding sperm pictures correlated with the DFI values measured by the SCSA technique [49]. Recently, AI assistance in CASA was used to evaluate the degree of chromatin dispersion in a large number of sperm with less inter-observer variability. A strong substantial association was found between the manual and AI-assisted results of DFI (r = 0.97, *p* < 0.001) [50]. However, despite the good diagnostic value, the spermatozoa analyzed using these advanced techniques cannot be selected or used in ART procedures. This is mainly because the fixation and staining processes limit cell viability or completely lyse the cells [49]. Hence, it is important to develop new AI-based algorithms to measure or predict DNA fragmentation levels in live spermatozoa without compromising their biological activity to increase the success rate of ICSI procedures [51].

A list of AI algorithms for predicting sperm DNA integrity or damage is presented in Table 4. Wang et al. (2019) detected SDF using a trained ML model that utilized the data from sperm pictures and images [52]. This comprehensive analysis indicated that morphology is associated with DNA integrity and can even predict DNA damage in individual spermatozoa [52]. These machine learning algorithms, once standardized, can uniquely assess the quality of individual sperm using photography and are free from any subjectivity. Large sperm imaging datasets with their corresponding DFI values are required to develop advanced AI- and ML-based algorithms to accurately predict the DFI of individual spermatozoa. Even with limited resources, comparable systems can be integrated into smart devices to enable patients to access these automated results at their convenience [28].

### 4.5. AI in Predicting Outcome of TESE

This surgical sperm retrieval procedure is mainly performed to isolate sperm from the testis of infertile men with nonobstructive azoospermia (NOA) for ART purposes, mainly IVF or ICSI. This procedure necessitates precise laboratory skills to isolate and identify spermatozoa in the biopsied testicular tissue. Recently, with the evolution of AI in medicine, new algorithms are being developed to predict the presence of sperm in testicular biopsies. A list of the AI algorithms associated with predicting the success of sperm retrieval is presented in Table 5. ML algorithms were able to forecast the likelihood of sperm retrieval success in NOA patients with 100% sensitivity, 69.2% specificity, and an AUC = 0.90 [53]. The more complex XGBoost ML algorithm was reported to have >90% sensitivity and >51% specificity in predicting the presence or absence of spermatozoa in patients with NOA [54]. ANN-based models were also developed using variables such as age; infertility duration; levels of male reproductive hormone such as LH, total and free testosterone, prolactin, and serum FSH; and right and left testicular volume to predict the presence of sperm cells in testes with an accuracy of 80.8% in NOA patients [55]. Similarly, another ANN model was able to correctly predict 59.4% of micro-TESE outcomes in patients using their preoperative clinical information [56].

Microscopic screening by expert personnel of the testicular biopsy samples to accurately identify sperm is a labor-intensive and time-consuming process. AI and ML were employed to generate algorithms that can identify the rare spermatozoa in the testicular biopsies without human intervention. A DNN-based deep object detection network was developed to identify sperm in testicular biopsy samples with a precision of 0.741 in real time [57]. The U-Net architecture, a CNN program, was trained with bright field images of spermatozoa from fertile males to detect rare sperm with an 84.4% PPV in biopsy samples that were clinically determined to be negative for spermatozoa [58]. All of these studies show that AI and ML have the potential to serve as support systems for physicians in decision making for performing TESE for their NOA patients.

**Table 5:** Artificial intelligence (AI) algorithms developed to detect sperm and predict success of testicular sperm extraction.

Studies	Dataset/Sample	Algorithm or Model	Performance or Outcomes
Bachelot et al., 2023 [53]	Semen	DNN	RF model: detected AUC = 0.90, sensitivity = 100%, specificity = 69.2%
Lee et al., 2022 [58]	Semen	CNN	For dissociated micro-TESE samples doped with an abundant quantity of sperm obtained: PPV = 84.0%, sensitivity = 72.7%, F1-score = 77.9% For dissociated micro-TESE samples doped with rare sperm obtained: PPV = 84.4%, sensitivity = 86.1%, F1-score = 85.2%
Wu et al., 2021 [57]	Semen	DNN	Obtained mean average precision (mAP) = 0.741, average recall (AR) = 0.376
Zeadna et al., 2020 [54]	Semen	GBTs	Detected AUC = 0.8, sensitivity = 91%, specificity = 25%
Ramasamy et al., 2013 [56]	Semen	ANN	Achieved ROC = 0.641, accuracy = 59.4%
Samli and Dogan 2004 [55]	Semen	ANN	Prediction accuracy = 80.80%

ANN—artificial neural network; AR—average recall; AUC—area under the curve; CNN—convolutional neural network; DNN—deep neural network; GBTs—gradient-boosted trees; mAP—mean average precision; PPV—positive predictive value; RF—random forest; ROC—receiver operating characteristic.

### 4.6. Strengths, Weaknesses, Opportunities, Threats (SWOT) Analysis of AI in Semen Analysis and Andrology Procedures

**Figure 2:**
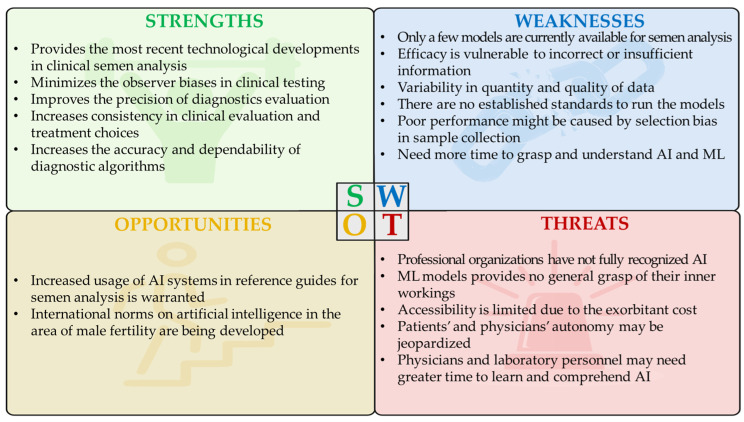
Strengths, weaknesses, opportunities, and threats (SWOT) analysis of AL and ML in andrology.

AI applications in semen analysis are still emerging. There are several potential limitations and pitfalls that are present in existing AI models. Standardized semen analysis requires a diverse dataset to train accurate models. The limited availability of high-quality, diverse semen samples can also hinder the development of robust AI models. Semen samples can vary significantly between individuals and even within the same individual over time. The complexity and variability of semen composition may pose challenges for AI models to generalize effectively. Furthermore, infertility is a complex issue, and semen analysis is just one aspect. AI models may not be able to address underlying causes of infertility that go beyond semen quality, such as hormonal imbalances or genetic factors. These shortcomings with the currently available AI models needs to be overcome to ensure their clinical usability in andrology laboratories for performing semen analysis. The strengths and weaknesses of AI in assisting semen analysis and other andrology procedures are summarized through a SWOT analysis (Figure 2).

## 5. Conclusions

We discussed the applications of several AI- and ML-based approaches in evaluating sperm parameters (concentration/count, motility, and morphology), sperm DNA integrity or damage, and success of TESE in NOA patients. AI tools have tremendous potential to manage large amounts of semen analysis data in order to improve the accuracy of the results. AI and ML algorithms trained with high-quality data could provide an advantage for andrologists and embryologists in creating standard, time-effective, and trustworthy sperm-selection procedures that improve embryo quality, pregnancy, and live-birth outcomes. While AI and ML hold promise in accurately assessing semen analysis and providing correct information to clinicians, it is essential to address challenges such as data quality, standardization, and ethical considerations. Additionally, the collaboration between ML experts and domain-specific medical professionals is crucial for the successful development and deployment of AI and ML applications in selecting a spermatozoon for ICSI. Over time, incorporating such approaches into medical practice would help reproductive healthcare professionals offer more effective and accurate diagnoses and patient care.

## Figures and Tables

**Table 1:** Artificial intelligence (AI) and machine learning (ML) algorithms used to evaluate sperm concentration or count.

Studies	Dataset/Sample	Algorithm or Model	Performance or Outcomes
Ory et al., 2022 [26]	Semen	Logistic regression, SVM and RF	Good predictive accuracy with AUC = 0.72
Lesani et al., 2020 [23]	Semen	FSNN, SPNN	Prediction accuracy: SPNN = 86%, FSNN = 93%
Tsai et al., 2020 [27]	Semen	Image recognition algorithm	AI algorithm vs. manual analysis: sperm concentration (r = 0.65, *p* < 0.001), motile sperm concentration (r = 0.84, *p* < 0.001)
Girela et al., 2013 [25]	Semen	ANN	Accuracy = 90%, sensitivity = 95.45%, specificity = 50%, PPV = 93.33%, NPV= 60%

ANN—artificial neural network; AUC—area under the curve; FSNN—full-spectrum neural network; NPV—negative predictive value; PPV—positive predictive value; RF—random forest; SPNN—selected peak neural network; SVM—support vector machine.

**Table 2:** Artificial intelligence (AI) and machine learning (ML) algorithms used to evaluate sperm motility.

Studies	Dataset/Sample	Algorithm or Model	Performance or Outcomes
Ottl et al., 2022 [32]	VISEM	SVR, MLP, CNN, RNN	Mean absolute error (MAE): SVR = 9.29, MLP = 9.50, CNN = 9.22, RNN = 9.86
Somasundaram and Nirmala 2021 [33]	Semen	THMA	Accuracy = 97.37%, with minimum execution time of 1.12 s.
Tsai et al., 2020 [27]	Semen	Bemaner AI algorithm	AI algorithm vs. manual analysis: r = 0.90, *p* < 0.001
Valiuškaitė et al., 2020 [34]	VISEM	CNN	MAE for predicted sperm motility = 2.92
Goodson et al., 2017 [29]	Semen	SVM	Accuracy = 89.9%
Girela et al., 2013 [25]	Semen	ANN	Accuracy = 82%, sensitivity = 89.29%, specificity = 43.75%, PPV = 89.29%, NPV = 43.75%

ANN—artificial neural network; CNN—convolutional neural network; MLP—multilayer perceptron; RNN—recurrent neural network; SVM—support vector machine; SVR—linear support vector regressor; THMA—tail-to-head movement algorithm.

**Table 3:** Artificial intelligence (AI) and machine learning (ML) algorithms used to evaluate sperm morphology.

Studies	Dataset/Sample	Algorithm or Model	Performance or Outcomes
Sato et al., 2022 [40]	JSD	DL	Abnormal sperm: sensitivity = 0.881 and PPV = 0.853 Normal sperm: sensitivity = 0.794 and PPV = 0.689
Abbasi et al., 2021 [41]	MHSMA	DTL DMTL	Detection accuracy: head = 84.0%, acrosome = 80.66%, and vacuole = 94.0%
Marín and Chang 2021 [35]	SCIAN-SpermSegGS	DL, U-Net, and Mask-RCNN	Dice coefficient using U-net with transfer learning: head = 0.96, acrosome = 0.94, and nucleus = 0.95
Nygate et al., 2020 [42]	Semen	DL, HoloStain	Virtual (holostain) vs. chemical staining: structural similarity (SSIM) = 0.85 ± 0.03
Valiuškaitė et al., 2020 [34]	VISEM	CNN	Accuracy of sperm head detection = 91.77%
Dubey et al., 2019 [20]	Semen	SVM	Accuracy = 89.93%, sensitivity = 91.18%, and specificity = 88.61%
Javadi and Mirroshandel 2019 [39]	MHSMA	DL	Detection accuracy: acrosome = 76.67%, head = 77.00%, vacuole = 91.33%
Movahed et al., 2019 [43]	SCIAN	CNN and SVM	Dice coefficient: head = 0.90, axial filament = 0.77, acrosome = 0.77, nucleus = 0.78, tail = 0.75, and mid-piece = 0.64
Riordon et al., 2019 [44]	HuSHeM and SCIAN	Deep-CNN, VGG16	Increased true positive rate: HuSHeM dataset = 94.1%, SCIAN dataset = 62%
Mirsky et al., 2017 [45]	Semen	SVM	Good accuracy with AUC = 89.59%
Shaker et al., 2017 [46]	SCIAN and HuSHeM	Dictionary learning	Detection accuracy: HuSeM dataset = 92%, SCIAN dataset = 62%
Shaker et al., 2016 [37]	Semen	Tail point algorithm	Dice coefficient accuracy: heads = 92%, acrosome = 84%, nucleus = 87%, and tail = 96%

AUC—area under curve; CNN—convolutional neural network; DL—deep learning; DTL—deep transfer learning; DMTL—deep multi-task transfer learning; HuSHeM—Human Sperm Head Morphology; JSD—Jikei sperm data set; MHSMA—Modified Human Sperm Head Morphology analysis; R-CNN—region-based convolutional neural network; SVM—support vector machine;.

**Table 4:** Artificial intelligence (AI) algorithms developed to measure or predict sperm DNA integrity or damage.

Studies	Dataset/Sample	Algorithm or Model	Performance or Outcomes
Kuroda et al., 2023 [50]	Semen	CNN	AI algorithm vs. manual scoring (r = 0.97, *p* < 0.001)
Noy et al., 2023 [51]	Semen	MobileNet CNN	Prediction accuracy = 90%, sensitivity = 0.93, specificity = 0.9
McCallum et al., 2019 [49]	Semen	Deep CNN	Sperm cell image vs. DNA quality (bivariate correlation ~0.43)
Wang et al., 2019 [52]	Semen	Logistic regression	Test accuracy = 82.7%

CNN—Convolutional Neural Network.

## Data Availability

Not applicable.

## Supplementary Materials

The following supporting information can be downloaded at: https://www.mdpi.com/article/10.3390/medicina60020279/s1, Table S1: Studies employing artificial intelligence (AI) or machine learning (ML) to evaluate or predict semen parameters, sperm DNA damage and outcome of surgical testicular sperm extraction (TESE).
