# Supplementary material for: Current Updates on Involvement of Artificial Intelligence and Machine Learning in Semen Analysis
Source: Medicina (Kaunas). 2024 Feb 6;60(2):279. doi: 10.3390/medicina60020279 (PMC10890589; doi:10.3390/medicina60020279)
Supplement: Supplementary file 1 [file medicina-60-00279-s001.zip › Supplementary Table S1.pdf]

Supplementary Table S1: Studies employing artificial intelligence (AI) or machine learning (ML) to evaluate or predict semen parameters, sperm DNA damage and outcome of surgical testicular sperm extraction (TESE).

Studies	Dataset/ Sample	AI/ ML Algorithm or Model	Parameter(s) Evaluated or Predicted
Kuroda et al., 2023 [50]	Semen	CNN	Sperm DNA fragmentation
Noy et al., 2023 [51]	Semen	CNN	Sperm DNA fragmentation
Bachelot et al., 2023 [53]	Semen	DNN	Predict success of sperm retrieval in men with NOA
Ory et al., 2022 [26]	Semen	Logistic regression, SVM and RF	Sperm concentration
Ottl et al., 2022 [32]	VISEM	SVR, MLP, CNN, RNN	Sperm motility
Sato et al., 2022 [40]	JSD	DL	Sperm morphology
Lee et al., 2022 [58]	Semen	CNN	Rare sperm detection in microTESE samples
Abbasi et al., 2021 [41]	MHSMA	DTL, DMTL	Sperm morphology
Marín & Chang 2021 [35]	SCIAN-SpermSegGS	DL, U-Net and Mask-RCNN	Sperm morphology
Somasundaram & Nirmala 2021 [33]	Semen	THMA	Sperm motility
Yüzkat et al., 2021 [36]	SMIDS, HuSHeM and SCIAN-Morpho	CNN	Sperm morphology
Wu et al., 2021 [57]	Semen	DNN	Sperm detection in microTESE samples
Iqbal et al., 2020 [42]	SCIAN and HuSHeM	CNN	Sperm morphology
Lesani et al., 2020 [23]	Semen	FSNN, SPNN	Sperm concentration
Nygate et al., 2020 [43]	Semen	HoloStain	Sperm morphology
Tsai et al., 2020 [27]	Semen	Image recognition algorithm and Bemaner AI algorithm	Sperm concentration and motility
Valiuškaitė et al., 2020 [34]	VISEM	CNN	Sperm motility and morphology

Zeadna et al., 2020 [54]	Semen	GBTs	Predict the presence or absence of spermatozoa in patients with NOA
Dubey et al., 2019 [20]	Semen	SVM	Sperm motility and morphology
Hicks et al., 2019 [31]	VISEM	CNN	Sperm motility
Javadi & Mirroshandel 2019 [39]	MHSMA	DL	Sperm morphology
McCallum et al., 2019 [49]	Semen	Deep-CNN	Sperm DNA fragmentation
Movahed et al., 2019 [44]	SCIAN	CNN and SVM	Sperm morphology
Riordon et al., 2019 [45]	HuSHeM and SCIAN	Deep-CNN	Sperm morphology
Wang et al., 2019 [52]	Semen	Linear and non-Linear Regression	Sperm DNA fragmentation
Chang et al., 2017 [38]	SCIAN	NN, NB, Decision trees, SVM	Sperm morphology
Goodson et al., 2017 [29]	Semen	SVM	Sperm motility
Mirsky et al., 2017 [46]	Semen	SVM	Sperm morphology
Shaker et al., 2017 [47]	SCIAN and HuSHeM	Dictionary learning	Sperm morphology
Shaker et al., 2016 [37]	Semen	Tail point algorithm	Sperm morphology
Girela et al., 2013 [25]	Semen	ANN	Sperm concentration
Ramasamy et al., 2013 [56]	Semen	ANN	Predict success of sperm retrieval in men with NOA before microTESE
Samli & Dogan 2004 [55]	Semen	ANN	Predict the presence or absence of spermatozoa in patients with NOA

ANN- Artificial Neural Network, CNN-Convolutional Neural Network, Deep CNN- Deep Conventional Neural Network, DL- Deep Learning, DNN-Deep Neural Networ, DTL- Deep Transfer Learning, DMTL- Deep Multi-task Transfer Learning, FSNN- Full Spectrum Neural Network, GBTs- Gradient-Boosted Trees, HuSHeM- Human Sperm Head Morphology, JSD- Jikei Sperm Data set, MHSMA- Modified Human Sperm Head Morphology analysis, MLP- Multilayer Perceptron, NOA- Non Obstructive Azoospermia, NN- Neural Network, NB- naive Bayes, R-CNN- Region Based Convolutional Neural Networks, RF- Random Forest, RNN- recurrent neural network, SPNN- Selected Peak Neural Network, SVM-Support Vector Machine, SVR- Linear Support Vector Regressor, THMA- Tail to Head movement algorithm
